# DLGR-1, a homolog of vertebrate DLGAP proteins, regulates spindle length and anaphase velocity during *C. elegans* meiosis

**DOI:** 10.17912/micropub.biology.001305

**Published:** 2024-08-16

**Authors:** Meghana Mahantesh Magadum, Francis McNally

**Affiliations:** 1 Molecular and Cellular Biology, University of California, Davis, Davis, CA, United States

## Abstract

Chromosome segregation requires a large number of microtubule-binding proteins that mediate spindle assembly and function during mitosis and meiosis. BLAST revealed a single *C. elegans* homolog of HURP/DLGAP5, a microtubule-binding protein that regulates mitotic and meiotic spindles in vertebrates. This homolog, W03A5.6 , was named DLGR-1 (DLGAP related). Time-lapse imaging of an endogenously tagged DLGR-1::GFP during *C. elegans* meiosis revealed plasma membrane localization specifically during anaphase I and anaphase II when the meiotic spindle is closely apposed to the plasma membrane. Time-lapse imaging of microtubules and chromosomes during meiosis in a strain with a CRISPR deletion of the DLGR-1 coding sequence revealed metaphase spindles that were significantly shorter than controls and chromosome separation velocities that were significantly slower than controls. Extrusion of chromosomes into polar bodies proceeded normally, consistent with the high progeny viability of the homozygous deletion strain. Thus DLGR-1 may play an accessory or redundant role in meiotic spindle function during *C. elegans* meiosis.

**Figure 1. Deletion of dlgr-1 results in shorter meiotic metaphase spindles and slower meiotic anaphase:**
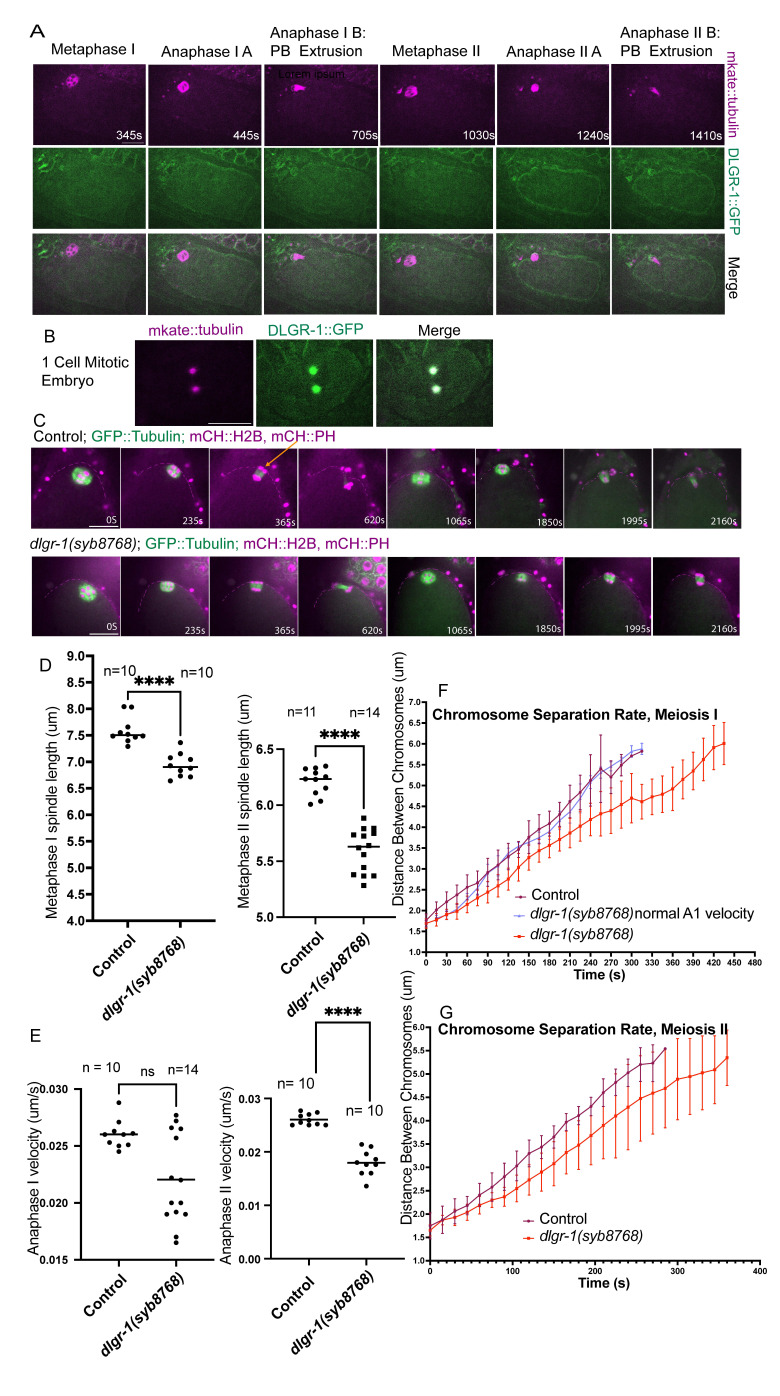
(A) Single-focal plane time-lapse images of meiosis in DLGR-1::AID::GFP, mKate::tubulin embryos. (B) Single-focal plane images of a 1-cell mitotic embryo with DLGR-1::AID::GFP, mKate::tubulin. (C) Single focal plane time-lapse images of meiosis in Control and *dlgr-1 ( syb8768 )* embryos expressing mCherry::histone H2B, mCherry::PH, GFP::tubulin. Magenta dotted lines emphasize the plasma membrane in the vicinity of the spindle and chromosomes. Orange arrow points to a protrusion in the plasma membrane which was used to determine the end of anaphase B. (D) Comparison of metaphase I and II spindle lengths in Control and *dlgr-1 ( syb8768 )* embryos. (E) Comparison of anaphase IB and anaphase IIB velocities in Control and *dlgr-1 ( syb8768 )* embryos.(F) Comparison of cumulative distance between chromosomes over time during anaphase B of meiosis I in Control and *dlgr-1 ( syb8768 )* embryos. (G) Comparision of cumulative distance between chromosomes over time during anaphase B of meiosis II in Control, *dlgr-1 ( syb8768 )* slow population, and *dlgr-1 ( syb8768 )* normal A1 velocity embryos. Statistics: Mann-Whitney U test (**p<0.01; ****p<0.0001). All scale bars = 10μm

## Description

Microtubule-based spindles mediate chromosome segregation during mitosis and meiosis. Spindle function is regulated by hundreds of proteins with many mechanistic details remaining to be elucidated. *C. elegans* is the only animal in which all aspects of meiotic spindle function can be monitored by time-lapse fluorescence microscopy within the intact mother. HURP (hepatoma upregulated protein)/DLGAP5 is a microtubule-binding protein that regulates spindle assembly and function in vertebrate cells during mitosis (Koffa et al., 2006; Silljé, Nagel, Körner, & Nigg, 2006; Tsou et al., 2003) and during mouse oocyte meiosis (Breuer et al., 2010) . A BLAST search of the *C. elegans* genome with the human HURP protein sequence revealed a single potential homolog, W03A5.6 . A reciprocal BLAST search of the human genome with the W03A5.6 protein sequence revealed significant homology in the C-terminal GKAP domain with human DLGAP1 to DLGAP5 but no striking homology with the N-terminal region of HURP/DLGAP5 which binds microtubules (Song, Craney, & Rape, 2014; Wong, Lerrigo, Jang, & Fang, 2008) . The *Drosophila* mars protein similarly only has homology with HURP in the GKAP domain (Yang et al., 2005) but still regulates mitotic spindle function (Zhang, Breuer, Förster, Egger-Adam, & Wodarz, 2009) . W03A5.6 shows the highest homology with DLGAP1/GKAP, a synaptic protein (Kim et al., 1997) that co-localizes with microtubules and recruits cytoplasmic dynein in migrating astrocytes (Manneville, Jehanno, & Etienne-Manneville, 2010) and binds dynein light chain in neurons (Moutin, Raynaud, Fagni, & Perroy, 2012) . Because of the limited homology, we refer to W03A5.6 as DLGR-1 (DLGAP related).

We first added auxin induced degron (AID) and green fluorescent protein (GFP) sequences to the endogenous DLGR-1 locus and monitored its localization during meiosis by time-lapse imaging. In 19 time-lapse sequences, DLGR-1::AID::GFP was concentrated at the plasma membrane/cortex during anaphase I ( Fig. 1A, 445s) and anaphase II ( Fig. 1A, 1240s), which were inferred from the length and orientation of the mKate::tubulin-labelled meiotic spindle. DLGR-1::AID::GFP also localized to the plasma membrane/cortex of oocytes in the gonad (n=7 worms) and to both plasma membrane and centrosomes in mitotic embryos ( Fig. 1B )(n=12 embryos).

Because neither auxin nor GFP(RNAi) reduced the fluorescence intensity of DLGR-1::AID::GFP, we generated a strain with a deletion of the entire coding sequence, *dlgr-1 ( syb8768 )* , to address function during meiosis. Meiosis proceeded relatively normally in *dlgr-1 ( syb8768 )* worms ( Fig. 1C ), hatch rates were similar to controls [ *dlgr-1 ( syb8768 )* 98% hatch, n=3 worms, n=635 progeny; FM1166 control 100% n= 3 worms, n= 745 progeny, p= .0004 Fisher's Exact Test], and the percentage of mitotic embryos with 2 polar bodies was not different than controls (control 100%, n=14; *dlgr-1 ( syb8768 )* 100%, n=10). However, meiotic metaphase spindles in *dlgr-1 ( syb8768 )* worms were shorter than controls ( Fig. 1D ) and meiotic anaphase chromosome separation velocities were slower than controls during anaphase II and in roughly half of anaphase I embryos ( Fig. 1E, F, G ).

The cortical localization of DLGR-1 suggests that it might influence anaphase velocities by scaffolding signaling molecules in close proximity to the spindle whereas the centrosome localization in mitotic embryos suggests that DLGR-1 might bind directly to microtubules of meiotic spindles at a level too low to observe with our imaging methods.

## Methods

CRISPR-mediated genome edits were performed by SUNY Biotech. For time-lapse imaging, anesthetized worms were mounted between an agarose pad and coverslip as described in (Danlasky et al., 2020) and subjected to single plane time-lapse imaging on a Yokogawa CSU-10 spinning disk confocal microscope equipped with an Olympus 100X 1.3 PlanApo objective and a Hammamatsu Orca Quest qCMOS detector. Exposures were captured every 5 seconds. Metaphase spindle length measurements were collected on spindles before the initiation of shortening. Anaphase B velocities were determined as described in (Li, Crellin, Cheerambathur, & McNally, 2023) .

## Reagents

**Table:** 

Strain number	Genotype	shorthand	Available from
FM1166	*ltSi1412 [pNA20; Pmex-5::mNeonGreen::tbb-2 operon linker mCh::his-11::Ptbb-2; cb-unc-119(+)]I; ItIs44 [pAA173; pie-1p-mCh::PH(PLC1delta1) + unc-119 (+)]V *	Control for PHX8768	fjmcnally@ucdavis.edu
PHX8768	*ltSi1412 [pNA20; Pmex-5::mNeonGreen::tbb-2 operon linker mCh::his-11::Ptbb-2; cb-unc-119(+)]I; dlgr-1 ( syb8768 ) III; ltIs44 [pAA173; pie-1p-mCh::PH(PLC1delta1) + unc-119 (+)]V *	*dlgr-1 ( syb8768 ) *	fjmcnally@ucdavis.edu
FM492	*wjIs76 [Cn_unc-119(+); pie-1p::mKate2:: tba-2 ]; cpIs103 [sun-1p::TIR1::F2A::mTagBFP2::degron-NLS::tbb-2 3'UTR]II *	Control for PHX7794	fjmcnally@ucdavis.edu
PHX7794	*wjIs76 [Cn_unc-119(+); pie-1p::mKate2::tba-2]; cpIs103 [sun-1p::TIR1::F2A::mTagBFP2::degron-NLS::tbb-2 3'UTR]II; dlgr-1 ( syb7794 [dlgr-1::AID::GFP]) III *	*dlgr-1 ::AID::GFP *	fjmcnally@ucdavis.edu
